# Efficacy and Safety of Remimazolam for Procedural Sedation: A Meta-Analysis of Randomized Controlled Trials With Trial Sequential Analysis

**DOI:** 10.3389/fmed.2021.641866

**Published:** 2021-07-27

**Authors:** Bo-Jyun Jhuang, Bo-Han Yeh, Yen-Ta Huang, Pei-Chun Lai

**Affiliations:** ^1^Department of Anesthesiology, Buddhist Tzu Chi General Hospital, Hualien, Taiwan; ^2^Department of Anesthesiology, Linkou Medical Center, Chang Gung Memorial Hospital, Taoyuan, Taiwan; ^3^Department of Surgery, National Cheng Kung University Hospital, College of Medicine, National Cheng Kung University, Tainan, Taiwan; ^4^Education Center, National Cheng Kung University Hospital, College of Medicine, National Cheng Kung University, Tainan, Taiwan

**Keywords:** remimazolam, endoscopy, procedural sedation, meta-analysis, trial sequential analysis

## Abstract

**Background:** Remimazolam is a new ultrashort-acting benzodiazepine. Remimazolam has been approved for procedural sedation by the US Food and Drug Administration in 2020. However, prior trials and the participants they enrolled were limited.

**Aim:** In this meta-analysis, we investigated the effectiveness and adverse events (AEs) of remimazolam during procedural sedation.

**Materials and Methods:** The study protocol was registered (doi: 10.37766/inplasy2020.8.0043), and six databases were searched. We performed meta-analysis, trial sequential analysis (TSA), and Grading of Recommendations, Assessment, Development, and Evaluation (GRADE) methodology for judging the certainty of evidence (CoE).

**Results:** A total of five randomized controlled trials with 1,248 participants were included. Compared with the use of midazolam, the utilization of remimazolam resulted in an increase in procedure success rate [odds ratio (OR) = 9.01, 95% confidence interval (CI): 2.35–34.57], a reduction in the application of rescue medication (OR = 13.58, 95% CI: 3.46–53.28), a decrease in time to recovery [minutes, weighted mean difference (WMD) = −5.70, 95% CI: −8.68 to −2.72], and a better cognitive recovery of Hopkins Verbal Learning Test-Revised (WMD = 5.22, 95% CI: 2.88–7.55). No difference was found in completion of procedure (OR = 1.68, 95% CI: 0.72–3.90) with inconclusive in TSA. Despite no difference of total AEs (OR = 0.60, 95% CI: 0.24–1.50), more detailed analysis of AEs remained inconclusive in TSA. The GRADE assessment demonstrated low to very low CoE.

**Conclusion:** Our analysis suggested that remimazolam may be a better choice for procedural sedation than midazolam. Nevertheless, further studies are warranted to conclusively establish its safety.

## Introduction

Endoscopic procedures, such as colonoscopy and bronchoscopy, are common for diagnostic and intervention purposes, and more than 18 million endoscopic procedures take place annually in the United States (1). Sedation is frequently used to minimize the endoscopic procedure-related anxiety, reduce the potential for injury during the procedures, and improve patient tolerability and satisfaction (2). Benzodiazepines are widely used for procedural sedation with minimal respiratory depression and hypotension. Diazepam and midazolam are the most used benzodiazepines for endoscopic sedation. Midazolam is frequently the drug of choice because of rapid onset, short duration of action, high potency, and lack of associated phlebitis (3). Midazolam has superior patient satisfaction with reduced respiratory depression compared with diazepam (4, 5). However, midazolam has minimal predictable post-procedural sedation due to the possible cumulative effects of its active metabolites (6).

Remimazolam is an ester-based benzodiazepine and can be rapidly hydrolyzed into inactive metabolites by tissue esterases (7). The onset of action of remimazolam is 1–3 min and has considerably short metabolic half-life (0.75 h), thereby providing adequate moderate sedation but faster recovery after intervention (8). The Food and Drug Administration has approved the BYFAVOTM (remimazolam) for the induction and the maintenance of procedural sedation in adults undergoing procedures lasting 30 min or less. Several clinical trials and reports about the sedation effects of remimazolam on endoscopic procedures are available, but studies and enrolled cases are limited. Moreover, systematic reviews and meta-analyses focusing on remimazolam are not available. Therefore, we have systematically reviewed randomized controlled trials (RCTs) to evaluate the effectiveness and safety issues of remimazolam in patients undergoing endoscopic procedures.

## Materials and Methods

### Protocol and Registration

Two independent investigators (BJ Jhuang, BH Yeh) systematically searched PubMed, Web of Science, Embase, Airiti Library, Google Scholar, and Cochrane Library from inception to May 31, 2021, without language limitation. The keywords searched using free texts and medical subject headings included “remimazolam,” “midazolam,” “safety,” “adverse event,” “efficacy,” “procedure,” “sedation,” “endoscopy,” “bronchoscopy,” and “colonoscopy.” In addition, we searched ClinicalTrials.gov and European Union Drug Regulating Authorities Clinical Trials Database for any unpublished or ongoing trial and additional data from published trials. The final list of included studies was decided by discussion among all authors with full agreement required before inclusion. We also reviewed the reference lists from original manuscripts, published reviews, systematic reviews, and meta-analyses to identify trials that were not listed in the original database. Our search strategy aimed to include every RCT that investigated the effectiveness of remimazolam on procedural sedation.

This study followed the latest statement of the Preferred Reporting Items for Systematic Reviews and Meta-Analysis (PRISMA 2020) (9). The report did not need ethics committee approval because raw data of human beings were not involved. We registered our protocols on the INPLASY with registration number of INPLASY202080043 (doi: 10.37766/inplasy2020.8.0043) and the protocol was updated and recorded the changes till June 13, 2021 (10).

### Study Selection

Titles and abstracts obtained from the initial literature search were screened independently by two authors (BJ Jhuang, BH Yeh). The authors also performed a full-text article review. Any discrepancy was resolved through group consensus. We enrolled the RCTs, in which interventions refer to subsequent or a single dose of remimazolam compared with midazolam groups on procedural sedation. We excluded studies with any of the following conditions: 1. review articles, case reports, or case series; 2. the included participants undergoing any endoscopic procedures with unclear anesthetics; 3. compared with any other anesthetics in the control group instead of midazolam; 4. unavailable data or without any relevant data for meta-analysis; 5. duplicated publications.

### Data Collection

Data were extracted and finalized from the eligible studies included by all authors. The data extracted from the eligible studies included demographic data, publication year, sample size, proportion of males, mean age, the American Society of Anesthesiologists physical (ASA) status, and funding sources. The primary outcomes included procedure success, completion of procedure, and no administration of rescue medication. Secondary outcomes were safety outcomes, including time to recovery, cognition recovery of Hopkins Verbal Learning Test-Revised (HVLT-R), and adverse events (AEs). Time to recovery was defined as time to first of three consecutive Modified Observer's Assessment of Alertness and Sedation (MOAA/S) scores of 5 after the end of the procedure. The MOAA/S scale is a measure of alertness/sedation and is derived from the original Observer's Assessment of Alertness/Sedation scale (11). The modified form was widely used for monitoring the sedation effect with the scale ranging from awake (5 point) to unresponsive (1 point). Cognition recovery was represented by change in Hopkins Verbal Learning Test-Revised (HVLT-R) (12). HVLT-R was administered by asking the patient to remember a list of 12 words read out by an investigator. The patient was then asked to recall as many as possible as he or she could immediately and at different times. This test was performed at baseline before any study drug and then within 5 min of becoming fully alert after endoscopy. Changes from before-dose to after-dose to total recall of the 12 words were compared between the remimazolam and midazolam groups. The risk of bias (RoB) was assessed by two authors (PC Lai and YT Huang) independently using the Risk-of-bias tool 2.0 (RoB 2.0) (13). Any assessment of RoB that may affect the cumulative evidence was also assessed by confirming the results by two authors (PC Lai and YT Huang) independently. Divergences were resolved by consensus. The results of RoB 2.0 were drawn using the “Risk-of-Bias Visualization tool” (14).

### Statistical Analysis

Dichotomous and continuous outcomes were presented as odds ratio (OR) and weighted mean difference (WMD), respectively, with 95% confidence intervals (CIs) (15). Statistical analysis was performed using the Microsoft Excel (Microsoft, Redmont, WA, USA) add-in MetaXL 5.3 (EpiGear International, Sunrise Beach, Australia) utilizing the inverse variance heterogeneity (IVhet) model (16) for dichotomous data. The random-effect model was used for analyzing the continuous data. Heterogeneities among studies were assessed using the I square (*I*^2^) statistics. An *I*^2^ higher than 50% represented substantial heterogeneity. For each outcome, we performed further subgroup analysis according to different procedure. To determine the subgroup of AEs, we analyzed the AEs of cardiovascular events (hypotension, hypertension, and bradycardia), respiratory events (decreased oxygen saturation), and neurological events (headache). As for the zero event, we further performed sensitivity analysis by Bayesian approach with Markov Chain Monte Carlo method (17). We used Microsoft-Excel-based NetMetaXL V.1.6.1 (Canadian Agency for Drugs and Technologies in Health, Ottawa, Canada) to perform WinBUGS 1.4.3 (MRC Biostatistics Unit, Cambridge, and Imperial College School of Medicine, London, UK) under the setting of with 150,000 simulations without zero correction and random-effect model with vague or informative prior (18). For publication bias, we presented the Doi plot with Luis Furuya-Kanamori (LFK) index for each endpoint (19). Values of LFK index outside the interval between −1 and +1 were defined as asymmetry of Doi plot, which may indicate publication bias.

### Trial Sequential Analysis

The TSA is used to approach and quantify the statistical reliability of data through repetitive and cumulative testing especially for meta-analyses (20). TSA was conducted using the TSA version 0.9.5.10 beta (Copenhagen Trial Unit, Center for Clinical Intervention Research, Rigshospitalet, Copenhagen, Denmark). Type I and Type II errors were set at 5 and 20%, respectively, in the model. The O'Brien–Fleming monitoring boundaries were applied for hypothesis testing. The cumulative effect of TSA was considered true positive if the Z curve crossed the O'Brien–Fleming monitoring boundaries and considered true negative if the Z curve entered the futility area. A total sample size that did not achieve the required information size (RIS) was defined as underpower. Random-effects model by using the Biggerstaff-Tweedie method was chosen (21). The calculated RIS considered the proportion of investigational and control events and the anticipated heterogeneity variance of the meta-analysis. The incidence of intervention and control arms was filled in the “overall events/total cases” of the enrolled studies. For continuous data, the “empirical” item or minimal difference was set for MD and variance, and the “model-based variance” item was applied.

### Grading of the Certainty of Evidence

We assessed every result by using the Grading of Recommendations, Assessment, Development, and Evaluation (GRADE) methodology (22). According to the GRADE methodology, the overall certainty of evidence (CoE) was judged by five downgrading and three upgrading domains. The level of CoE was classified as high, moderate, low, or very low. Grading was made using the GRADEpro software (available from gradepro.org).

## Results

**Figure 1:**
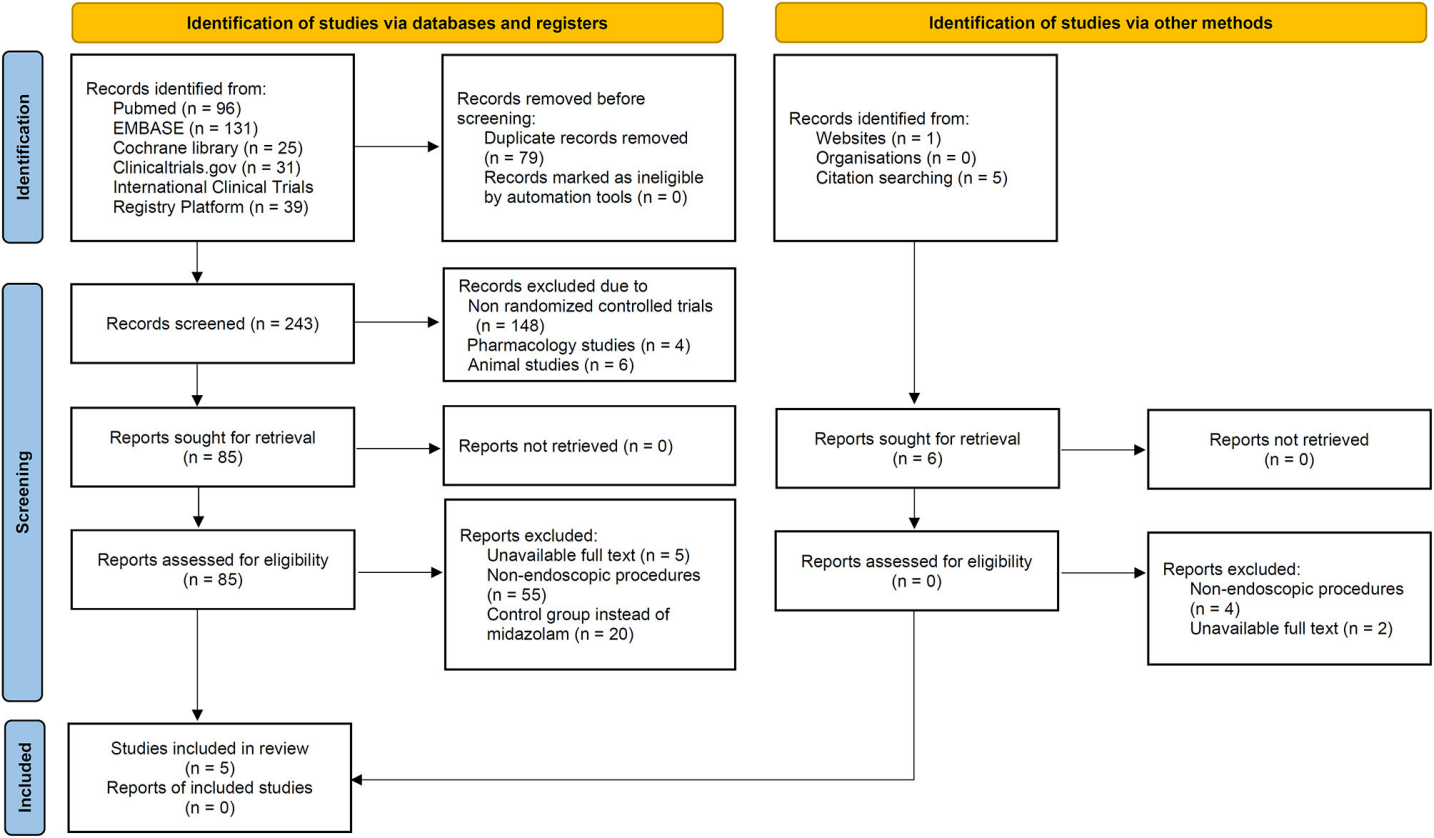
Flow diagram of preferred reporting items for systematic reviews and meta-analysis (PRISMA) 2020.

A total of 322 articles were identified from the primary electronic databases [PubMed, 96; EMBASE, 131; Cochrane library, 25; ClinicalTrials.gov, 31; International Clinical Trials Registry Platform (ICTRP), 39]. In the search of gray literature, 6 records identified via websites (*n* = 1), citation searching (*n* = 5). A total of 79 studies were excluded due to duplication, 148 studies due to non-RCT, 4 studies due to pharmacology studies, 6 studies due to animal studies, 7 studies with unavailable full text, 59 studies due to non-endoscopic procedures and 20 studies due to control group instead of midazolam. Finally, five articles were included in the meta-analysis. The results were demonstrated as PRISMA (Preferred Reporting Items for Systematic Reviews and Meta-Analyses) flowchart (Figure 1) according to the PRISMA 2020 statement (9).

Table 1 demonstrated the characteristics of enrolled RCTs. The five included studies involved 1,248 participants. All studies were RCTs and conducted in the United States. The age of participants ranged from 18 to 95 years, and the proportion of males ranged from 45 to 55.8%. One trial (23) enrolled patients with an ASA status of 1 and 2, one trial (27) enrolled patients with an ASA status of 3 and 4, and three other trials (24–26) enrolled patients with an ASA status of 1, 2, and 3. The types of procedure included colonoscopy (24, 25, 27), UGI endoscopy (23), and bronchoscopy (26). All enrolled studies were all funded by PAION, UK Ltd.

**Table 1:** Characteristics of included trials.

**Trials**	**Funding**	**Age (yr) mean (±SD)**	**Male (%)**	**Procedures**	**Country**	**ASA physical status**	**Sample size**	**Intervention/comparator**	**Main outcome measures**
Borkett et al. (23) NCT00869440	PAION UK Ltd.	41 (±13.86)	46	UGI endoscopy	USA	1, 2	100	1. Remimazlolam 0.10 mg/kg 2. Remimazlolam 0.15 mg/kg 3. Remimazlolam 0.20 mg/kg 4. Midazolam 0.075 mg/kg	1. Procedure success 2. Completion of procedure 3. No administration of rescue medication 4. Time to recovery 5. Adverse events 6. Cognition recovery of HVLT-R
Pambianco et al. (24) NCT01145222	PAION UK Ltd.	54.6 (±9.02)	45	Colonoscopy	USA	1, 2, 3	162	1. Remimazlolam 8.0/3.0 mg 2. Remimazlolam 7.0/2.0 mg 3. Remimazlolam 5.0/3.0 mg 4.Midazolam 2.5/1.0 mg	1. Procedure success 2. Completion of procedure 3. No administration of rescue medication 4. Time to recovery 5. Adverse events 6. Cognition recovery of HVLT-R
Rex et al. (25) NCT02290873	PAION UK Ltd.	54.4 (±10.12)	49.7	Colonoscopy	USA	1, 2, 3	461	1. Remimazolam 5.0 mg 2. Placebo 5.0 mg 3. Midazolam 1–1.75 mg	1. Procedure success 2. Completion of procedure 3. No administration of rescue medication 4. Time to recovery 5. Adverse events
Pastis et al. (26) NCT02296892	PAION UK Ltd.	62 (±12)	46	Bronchoscope	USA	1, 2, 3	446	1. Remimazolam 5.0 mg 2. Placebo 5.0 mg 3. Midazolam 1–1.75 mg	1. Procedure success 2. Completion of procedure 3. No administration of rescue medication 4. Time to recovery 5. Adverse events
Rex et al. (27) NCT02532647	PAION UK Ltd.	62.5 (±9.32)	55.8	Colonoscopy	USA	3, 4	79	1. Remimazolam 2.5–5.0 mg 2. Placebo 3. Midazolam 1.0 mg	1. Procedure success 2. Time to Start of Procedure 3. Time to recovery 4.Adverse events

*UGI, upper gastrointestinal; HVLT-R, Hopkins Verbal Learning Test-Revised*.

### RoB Assessment

**Figure 2:**
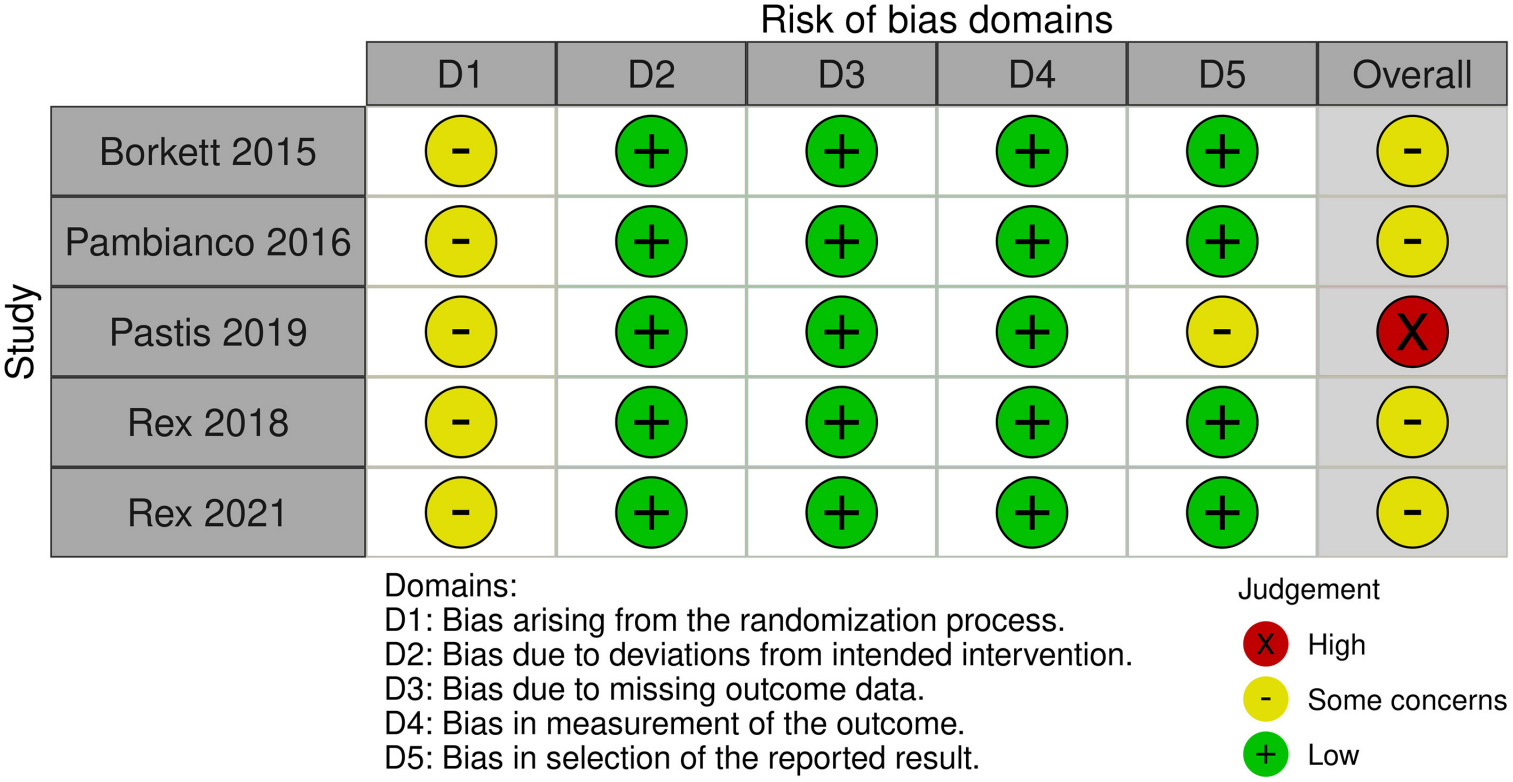
Risk-of-bias assessment.

The overall RoB of the five enrolled studies were judged as “some concerns” in four trials (23–25, 27) and high in one trial (26) (Figure 2). In the domain of randomization, five enrolled trials were all judged as “some concerns” because the allocation concealment was not clearly described. As for the domain of deviation from the intended intervention, missing outcome data, and measurement of outcome, the five enrolled trials had low RoB. However, we compared the results of the trial from the ClinicalTrials.gov of NCT02296892 (28) and the published article (26). The reported rate of AEs showed significant difference in the remimazolam (88.45 vs. 34.7%) and the midazolam (91.30 vs. 31.9%) groups. Given the difference in reporting AEs, the domain of selective reporting bias was judged as “some concerns” in the trial reported by Pastis et al. Finally, the overall RoB was judged as high for this trial.

### Outcomes

#### Primary Outcomes: Procedure Success (Five Trials)

**Figure 3:**
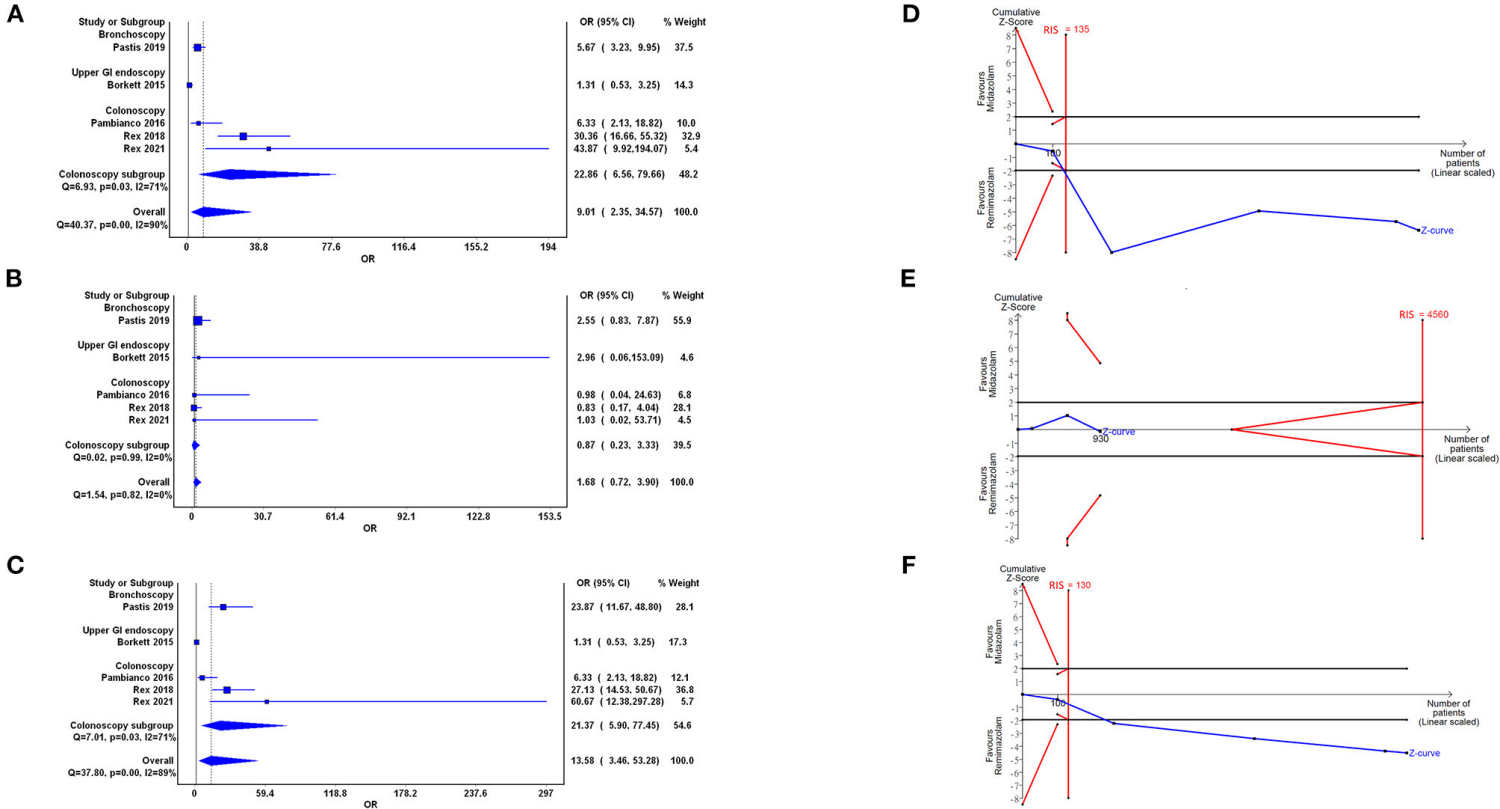
Forest plot **(A–C)** and trial sequential analysis **(D–F)** of procedure success **(A,D)**, completion of procedure **(B,E)**, and no administration of rescue medication **(C,F)** between remimazolam and midazolam, respectively. OR, odds ratio; CI, confidence interval; RIS, required information size.

Based on the pooling of data from five trials with 1,093 participants, our analyses showed statistical difference between the use of remimazolam and midazolam on procedure success (OR = 9.01, 95% CI: 2.35–34.57). Heterogeneity among all studies was high with *I*^2^ of 90% (Figure 3A). We further examined the results by using TSA. The cumulative Z Curve showed that the sample size was adequate, and the traditional boundaries were crossed since the first two studies, indicating a true positive result (Figure 3D). Results indicated that remimazolam was significantly superior to midazolam in the outcome of procedure success under the indication of procedural sedation. Doi plot yielded minor asymmetry with LFK index of 1.36 (Supplementary Figure 1A). We further performed subgroups analysis. In the subgroup of colonoscopy, the OR of procedural success of remimazolam to midazolam was 22.86 (95% CI: 6.56–79.66, *I*^2^: 71%), which remained statically significant.

#### Primary Outcomes: Completion of Procedure (Five Trials)

Our analysis showed no statistically significant difference in the completion of procedure in the use of remimazolam compared with the use of midazolam (OR = 1.68, 95% CI: 0.72–3.90) with low heterogeneity (*I*^2^ = 0%; Figure 3B). The cumulative Z curve in TSA neither crossed the O'Brien–Fleming monitoring boundaries nor enter the inner wedge of futility borders, which indicated an inconclusive result (Figure 3E). Doi plot yielded major asymmetry with LFK index of 2.31 (Supplementary Figure 1B).

#### Primary Outcomes: No Administration of Rescue Medication (Five Trials)

Five trials demonstrated that the rescue medication usage in the remimazolam group was significantly reduced compared with that in the midazolam group (OR = 13.58, 95% CI: 3.46–35.28, *I*^2^ = 89%; Figure 3C). The cumulative Z curve in TSA showed that the sample size was adequate and that the Z curve crossed traditional boundaries since the first two studies, indicating the true positive result in the favor of remimazolam usage (Figure 3F). Doi plot yielded major asymmetry with LFK index of 2.28 (Supplementary Figure 1C). In the subgroup analysis of the colonoscopy, the OR of this outcome between remimazolam and midazolam group was 21.37 (95% CI: 5.90–77.45, *I*^2^: 71%).

#### Secondary Outcomes: Time to Recovery (Five Trials)

**Figure 4:**
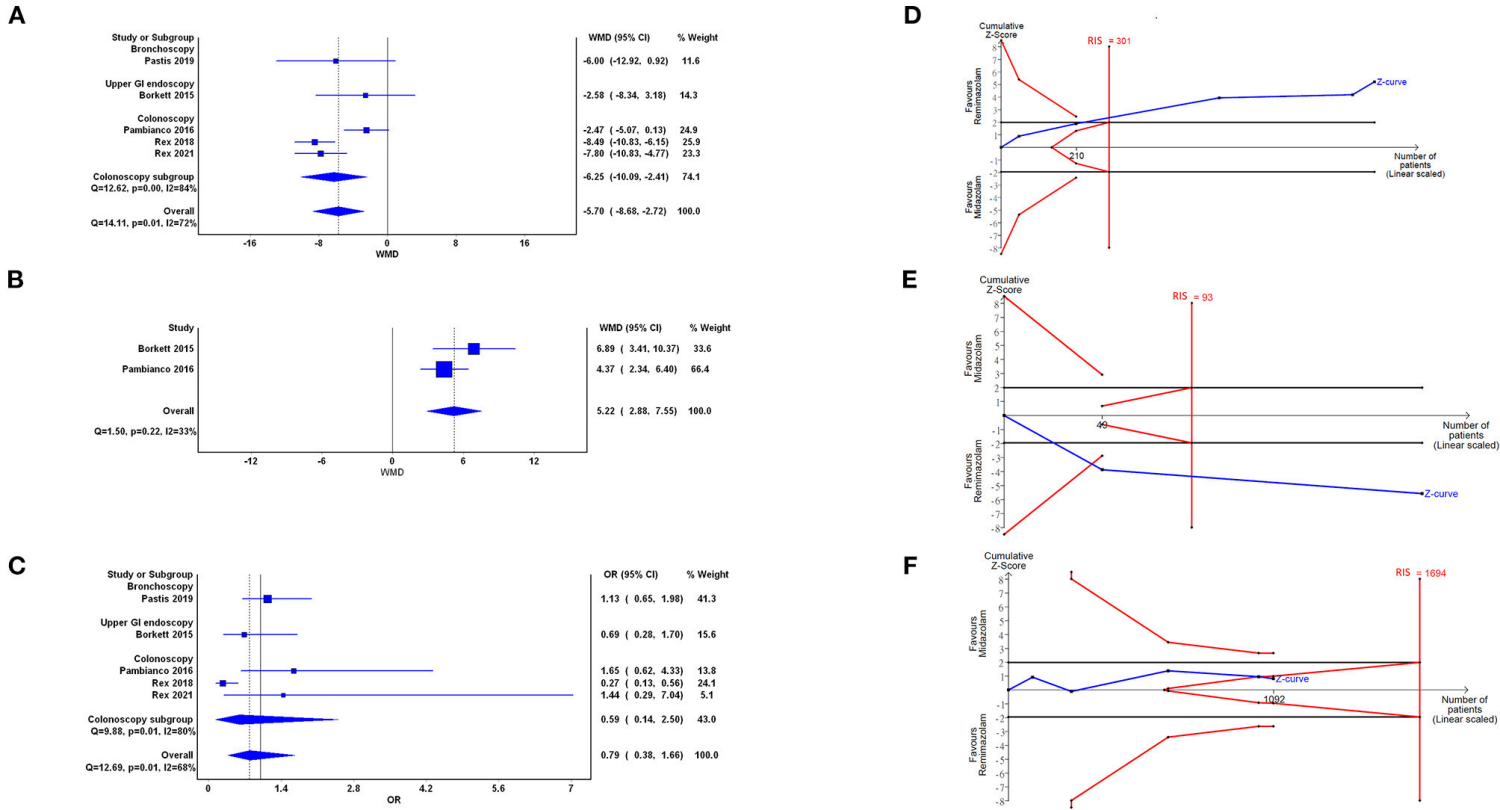
Forest plot **(A–C)** and trial sequential analysis **(D–F)** of time to recovery **(A,D)**, cognition recovery of Hopkins Verbal Learning Test-Revised (HVLT-R; **B,E**), and adverse events (AEs; **C,F**) between remimazolam and midazolam, respectively. OR, odds ratio; WMD, weighted mean difference; CI, confidence interval; RIS, required information size.

Five trials with 1,041 participants demonstrated that the time to recovery significantly reduced with the use of remimazolam compared with the use of midazolam (WMD = −5.70, 95% CI: −8.68 to −2.72, *I*^2^ = 72%; Figure 4A). The cumulative Z curve crossed the O'Brien–Fleming boundaries, which indicated true positive result and adequate sample size (Figure 4D). Doi plot yielded major asymmetry with LFK index of 2.31 (Supplementary Figure 1D). In the subgroup analysis of colonoscopy, the WMD was −6.25 (95% CI: −10.09 to −2.41, *I*^2^: 84%).

#### Secondary Outcomes: Change in HVLT-R (Two Trials)

Two trials (23, 24) with 209 participants demonstrated better cognitive recovery in the remimazolam group compared with the midazolam group (WMD = 5.22, 95% CI: 2.88–7.55, *I*^2^ = 33%; Figure 4B). TSA showed that the cumulative Z curve crossed the traditional boundaries and the cases of included trials were more than the RIS. Doi plot were not able to be presented when only two studies were enrolled. The above results indicated significantly true positive result with conclusion that remimazolam was superior to midazolam (Figure 4E).

#### Secondary Outcomes: AEs (Five Trials)

Five trials with 1,091 participants demonstrated that no statistical difference between the remimazolam and the midazolam groups in episode of AEs (OR = 0.79, 95% CI: 0.38–1.66, *I*^2^ = 68%; Figure 4C). The cumulative Z curve in TSA crossing into the futility area indicated a conclusion of indifference between 2 groups (Figure 4F). The cumulative Z curve did not cross the line of RIS, which may downgrade the CoE in the domain of imprecision. Doi plot yielded minor asymmetry with LFK index of 1.36 (Supplementary Figure 1E). As for more detailed AEs, Supplementary Figure 2 showed that there was no statistically difference of remimazolam and midazolam in (A) decreased oxygen saturation (OR = 1.15, 95% CI: 0.65–2.03), (B) headache (OR = 1.10, 95% CI: 0.35–3.52), (C) hypotension (OR = 0.61, 95% CI: 0.29–1.28), (D) hypertension (OR = 0.99, 95% CI: 0.68–1.43), and (E) bradycardia (OR = 0.65, 95% CI: 0.38–1.12). The TSA results of above detailed AEs were shown as Supplementary Figures 2F–L, respectively. Only in the reported AEs of hypotension (panel J), the cumulative Z curve in TSA crossed the RIS. There was one zero event presented in the AE with “bradycardia” in the midazolam group, so we further performed sensitivity analysis by random-effect model Bayesian approach. The OR of bradycardia with vague and informative prior showed 0.71 [95% credible interval (CrI): 0.22–2.89] and 0.68 (95% CrI: 0.39–1.22), respectively (Supplementary Figure 3).

### GRADE Assessment

The GRADE assessment demonstrated an overall low CoE in the outcomes of procedure success, no administration of rescue medication, and HVLT-R. In addition, overall very low CoE was ranked in the outcomes of completion of procedure, time to recovery, and AEs (Table 2). We downgraded the overall CoE in the domain of RoB, imprecision, and publication bias. The serious study-limitation was concerned because all enrolled articles were judged as “some concern” in overall RoB. Although high heterogeneity was found in some endpoints (procedure success, no administration of rescue medication, and time to recovery), the result in each individual study faced the same direction in each endpoint. Therefore, we did not rate down the CoE in the domain of inconsistency. Serious imprecision in the outcome of completion of procedure and AEs was defined by the pooled cases do not fill with the number of RIS in TSA. The publication bias was presented in all endpoints because asymmetry was observed in all Doi plots and enrolled studies were all funded by PAION, UK Ltd with limited sample sizes (29).

**Table 2:** GRADE assessment.

**Remimazolam compared to midazolam for procedural sedation**							
**Certainty assessment**							**Summary of findings**
**Participants (studies) Follow up**	**Risk of bias**	**Inconsistency**	**Indirectness**	**Imprecision**	**Publication bias**	**Overall certainty of evidence**	**Anticipated absolute effects Risk difference**
**Procedure success**							
1091 (5 RCTs)	Serious^a^	Not serious	Not serious	Not serious	Publication bias strongly suspected ^b^	⊕⊕◯◯ LOW	**478 more per 1,000** (from 209 more to 598 more)
**Completion of procedure**							
1091 (5 RCTs)	Serious^a^	Not serious	Not serious	Serious^c^	Publication bias strongly suspected^b^	⊕◯◯◯ VERY LOW	**10 more per 1,000** (from 10 fewer to 19 more)
**No administration of rescue medication**							
1091 (5 RCTs)	Serious^a^	Not serious	Not serious	Not serious	Publication bias strongly suspected^b^	⊕⊕◯◯ LOW	**482 more per 1,000** (from 293 more to 535 more)
**Time to recovery**							
1041 (5 RCTs)	Serious ^a^	Not serious	Not serious	Not serious	Publication bias strongly suspected^b^	⊕⊕◯◯ LOW	MD **5.7 fewer** (8.68 fewer to 2.72 fewer)
**Change in cognition recovery of Hopkins Verbal Learning Test-Revised**							
209 (2 RCTs)	Serious^a^	Not serious	Not serious	Not serious	Publication bias strongly suspected^b^	⊕⊕◯◯ LOW	MD **5.22 higher** (2.88 higher to 7.55 higher)
**Adverse event**							
1091 (5 RCTs)	Serious^a^	Not serious	Not serious	Serious^c^	Publication bias strongly suspected^b^	⊕◯◯◯ VERY LOW	**58 fewer per 1,000** (from 237 fewer to 113 more)

a *Overall risk of bias by Cochrane Rob 2.0 tool was judged as “some concern”*.

b *Asymmetry in Doi plot, and all commercially funded by PAION, UK Ltd with small sample sizes*.

c *Insufficient sample size, calculated by trial sequential analysis*.

## Discussion

For the moderate procedural sedation, such as UGI endoscopy and colonoscopy, benzodiazepine, especially midazolam, remains to be the drug of choice in sedatives according to the American Society of Anesthesia guidelines (30). However, increased anterograde amnesia has been reported in the midazolam group compared to other medications in a publication of Cochrane Database Systematic Review (31). Recent systematic reviews and meta-analyses also focused on comparing midazolam and propofol in procedural sedation (32–34). Although propofol has some advantage of shorter recovery time, propofol-related hypotension remains an important issue. It seems necessary to find out a new candidate medication for procedural sedation with better efficacy and safety than current standard choices. This study provided some potential benefits of remimazolam as a qualified drug for procedural sedation in the future.

Remimazolam is a benzodiazepine developed to provide sedative effects with an ultrashort half-life (8), translating into a quick onset and offset of effect. One of the potential benefits of remimazolam over midazolam comes from the design of the molecule. Remimazolam is rapidly degraded into an inactive metabolite by ubiquitous tissue esterases and is not metabolized by cytochrome-dependent hepatic pathways (7). Like other benzodiazepines, remimazolam can be reversed with flumazenil to terminate sedation or anesthesia. Therefore, remimazolam seems a good candidate for procedural sedation based on abovementioned properties of pharmacodynamics and pharmacokinetics. Remimazolam has been approved on 23 January 2020 in Japan for use in general anesthesia and on 5 July 2020 in the United States for procedural sedation (35). For procedural sedation, RCTs have only compared remimazolam with midazolam as abovementioned studies or propofol (36, 37). To determine the beneficial evidence of remimazolam compared to current standard regiments, more large-scale RCTs are warranted. During the waiting period for future RCTs, meta-analysis with trial sequential analysis may be good tools to determine the pooled effects from the past trials.

This study is the first report to compare remimazolam to midazolam for procedural sedation by pooled estimates of meta-analyses. As for the primary outcomes, the effect of remimazolam in procedure success and no need for administration of rescue medication has a number-needed-to-treat of 2 and 2, respectively. Moreover, our analysis shows a significant reduction in the time to recovery and better cognitive recovery scored by HVLT-R after remimazolam administration compared with those after midazolam administration, which can be attributed to less rescue medication needed and less time needed to regain full alertness. These results may be explained by the difference of mean terminal elimination half-life between remimazolam and midazolam (~0.75 and 4 h, respectively) (7, 38). These findings result in a rapid and predictable recovery of patients from their sedation and benefit the patients by potentially being able to leave the unit quickly and perform everyday tasks rapidly in the gastroenterology suite (23) and bronchoscopy laboratories (26). From the meta-analyses of sparse studies, remimazolam may be both effective and efficient drug for procedure sedation. Although our pooled results concluded no statistical difference of AEs between the remimazolam and the midazolam groups via TSA, we realized that the variety of AEs may indicate different meanings and importance, so we also calculated most frequent reported five AEs individually. Due to currently limited data, result of individual AEs remained inconclusive. Although obvious and significant difference in comparison of remimazolam and midazolam was observed in many endpoints, the CoE based on the GRADE methodology demonstrates only low to very low. Only enrolling more rigorous RCTs with numerous cases can upgrade the CoE in the future.

In this study, we performed some statistical methods for the further confirmation of precise results. We used the IVhet model for minimized the possibility or under- or over-estimate the heterogeneity by traditional random or fixed effect setting in meta-analyses with sparse studies (16). Besides, we applied TSA and Doi plot, as assistant tools for advanced and rigorous rating of CoE in GRADE. One study reported that TSA adoption would lead to a more frequent downgrading of the CoE and could be a potential supplement for a more thorough assessment of imprecision in GRADE (39). Recently Jia et al. demonstrated that many meta-analyses of rare events were underpowered, and the individual AEs in our meta-analyses were consistent with this observation based on the results of TSA (40). In contrast, Z curves of many endpoints crossed the O'Brien–Fleming boundaries and the lines of RIS early only after accumulation of the first two studies, and we thought these results might also hints of effective and efficient properties of remimazolam for procedure sedation. We preferred using Doi plot because the LFK index of Doi plot demonstrated a higher sensitivity to judge publication bias, compared with Egger's regression test when fewer studies were enrolled (19). Xu et al. highly recommended using a proposed framework when dealing with zero event in meta-analysis, including a conduct of sensitivity analysis using other available statistical methods for duplicate confirmation (17). Due to the zero event in the single arm of midazolam group in the AEs of bradycardia, we decided to use Bayesian approach, one of the recommended methodology, to test whether the results were robust (17, 41). Consistent results were observed between frequentist approach with IVhet model and Bayes' theorem with vague or informative prior.

In conclusion, this meta-analysis has systematically reviewed the RCTs to clarify the effects of remimazolam in procedural sedation. The pooled data show statistically significant differences in the outcome of procedure success. The use of remimazolam has resulted in less administration of rescue medication, less time to recovery, and better cognitive recovery compared with the use of midazolam during procedural sedation. We rated the evidence, in the main, as being of low to very low quality based on standard methodology of GRADE. Under the consideration the potential benefit and harm conclusively, we give a conditional recommendation for the use of remimazolam in procedural sedation based on the pooled estimates of good effect and acceptable safety. For determining the evidence of such a new drug, more RCTs with updated meta-analyses is warranted.

## Limitation

Many of the limitations of this study were already examined in the discussion. The main limitations of this study were the limited numbers of studies and participants. Only five trials were included in this meta-analysis. Thus, the conclusion might not be definitive. Nevertheless, the results of TSA showed that several outcomes were with an adequate sample size and statistical significance.

## Conclusion

We clarified the effects of the newest benzodiazepine, namely, remimazolam, on procedural sedation via meta-analysis and TSA of RCTs. The pooled data showed that the use of remimazolam resulted in a higher rate of procedure success, lesser administration of rescue medication, shorter time to recovery, and better cognitive recovery than the use of midazolam during procedural sedation. However, this meta-analysis did not conclusively establish the safety of remimazolam. Despite there was no difference of total AEs between remimazolam and midazolam group, more detailed analysis of AEs yielded inconclusive results. Nevertheless, our study was the first to compare the utilization of remimazolam and midazolam for procedural sedation by pooling RCTs with small samples. The endpoints with statistical significance still demonstrated the potential clinical applications of remimazolam in procedural sedation.

## Data Availability Statement

The original contributions presented in the study are included in the article/Supplementary Material, further inquiries can be directed to the corresponding author/s.

## Author Contributions

Y-TH and P-CL: conceptualization, validation, and formal analysis. B-JJ and B-HY: methodology, software, and writing of the original draft of the manuscript. P-CL: data curation, reviewing and editing of the manuscript, and supervision. Y-TH: visualization. All authors have read and agreed to the published version of the manuscript.

## Conflict of Interest

The authors declare that the research was conducted in the absence of any commercial or financial relationships that could be construed as a potential conflict of interest.

## Publisher's Note

All claims expressed in this article are solely those of the authors and do not necessarily represent those of their affiliated organizations, or those of the publisher, the editors and the reviewers. Any product that may be evaluated in this article, or claim that may be made by its manufacturer, is not guaranteed or endorsed by the publisher.
