# Supplementary material for: Efficacy and Safety of Remimazolam for Procedural Sedation: A Meta-Analysis of Randomized Controlled Trials With Trial Sequential Analysis
Source: Front Med (Lausanne). 2021 Jul 27;8:641866. doi: 10.3389/fmed.2021.641866 (PMC8353129; doi:10.3389/fmed.2021.641866)

Supplemental Figure 1. Doi plot of (A) procedure success, (B) completion of procedure, (C) no administration of rescue medication, (C) time to recovery, and (E) adverse events. LFK, Luis Furuya-Kanamori; OR, odds ratio; ln, natural logarithm.


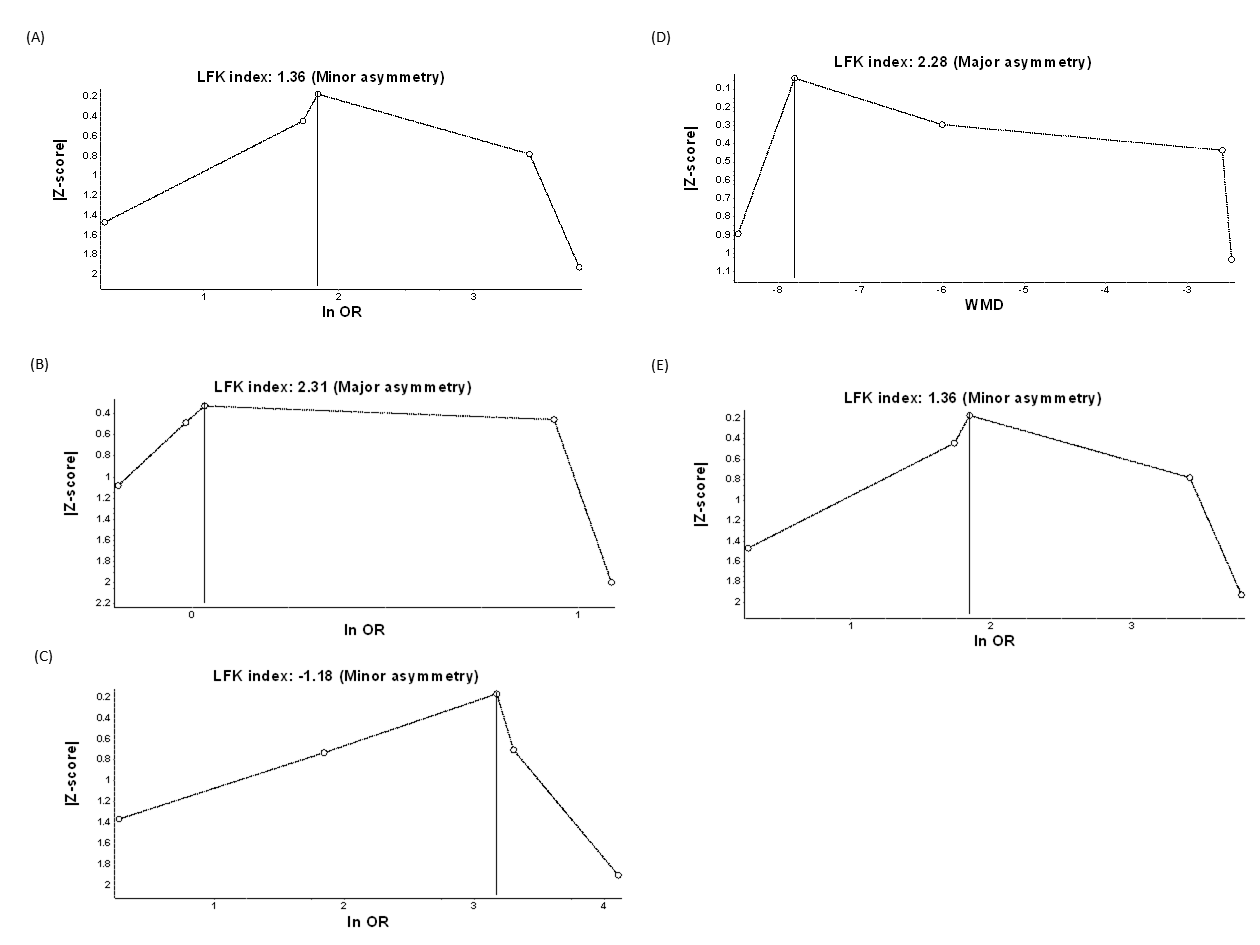


Supplemental Figure 2. Forest plot (A-E) and trial sequential analysis (F-L) of decreased oxygen saturation (A & F), headache (B & I), hypotension (C & J). hypertension (D & K), and bradycardia (E & L) between remimazolam and midazolam, respectively. OR, odds ratio; CI, confidence interval; RIS, required information size.


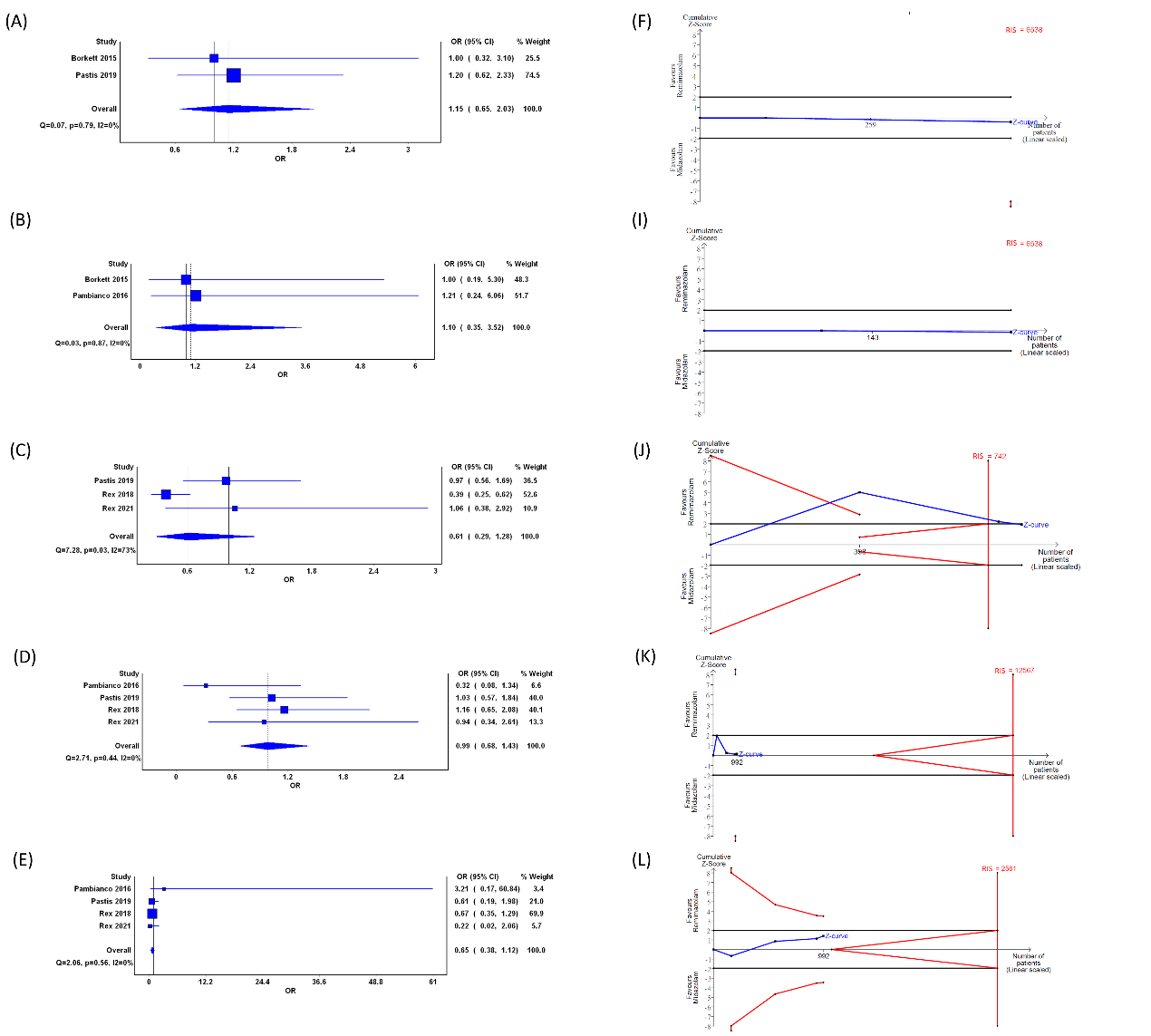


Supplemental Figure 3. Meta-analysis of bradycardia between remimazolam and midazolam using random-effect model Bayesian approach with vague or informative prior. OR, odds ratio; CrI, credible interval.


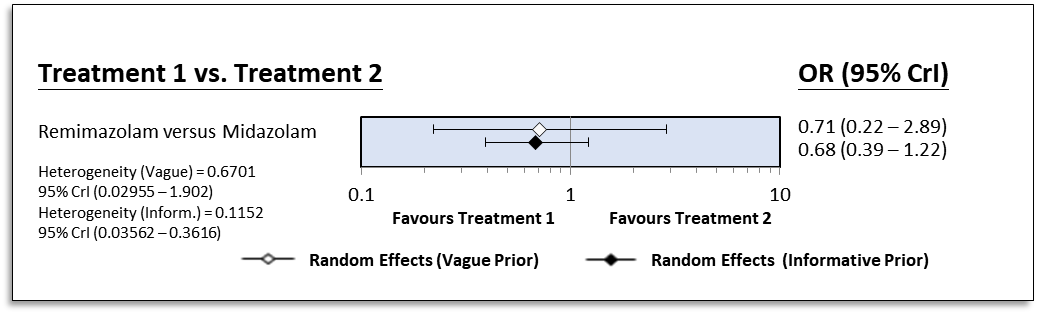

Supplement: Supplementary file 1 [file Data_Sheet_1.docx]
